# The Keeley Craze

**Published:** 1892-06

**Authors:** 


### THE KEELEY CRAZE.

The astonishing eagerness with which the medically ignorant public run after every *ignis fatuus* launched by pretentious charlatans, is a source of a sort of grim amusement to educated medical men. No better illustration of popular gullibility ever occurred than in the present popularity of the scheming Keeley, of Dwight. The ignorance of the man is aptly displayed in a recent article published in a Chicago journal, in which scientific terms are treated in a most reckless manner, and in a way calculated to excite the derision of every reader having a modicum of scientific knowledge. A few weeks ago the sage of Dwight made the ridiculous assertion that 15 grains of asafetida would cure any case of La Grippe, and the demand for asafetida pills straightway became enormous. In the course of a few weeks the present mania will be recovered from and a new one will break out.—*Good Health.*

